# FPGA acceleration of the phylogenetic likelihood function for Bayesian MCMC inference methods

**DOI:** 10.1186/1471-2105-11-184

**Published:** 2010-04-12

**Authors:** Stephanie Zierke, Jason D Bakos

**Affiliations:** 1 Department of Computer Science and Engineering, University of South Carolina, Columbia, SC, USA

## Abstract

**Background:**

Likelihood (ML)-based phylogenetic inference has become a popular method for estimating the evolutionary relationships among species based on genomic sequence data. This method is used in applications such as RAxML, GARLI, MrBayes, PAML, and PAUP. The Phylogenetic Likelihood Function (PLF) is an important kernel computation for this method. The PLF consists of a loop with no conditional behavior or dependencies between iterations. As such it contains a high potential for exploiting parallelism using micro-architectural techniques. In this paper, we describe a technique for mapping the PLF and supporting logic onto a Field Programmable Gate Array (FPGA)-based co-processor. By leveraging the FPGA's on-chip DSP modules and the high-bandwidth local memory attached to the FPGA, the resultant co-processor can accelerate ML-based methods and outperform state-of-the-art multi-core processors.

**Results:**

We use the MrBayes 3 tool as a framework for designing our co-processor. For large datasets, we estimate that our accelerated MrBayes, if run on a current-generation FPGA, achieves a 10× speedup relative to software running on a state-of-the-art server-class microprocessor. The FPGA-based implementation achieves its performance by deeply pipelining the likelihood computations, performing multiple floating-point operations in parallel, and through a natural log approximation that is chosen specifically to leverage a deeply pipelined custom architecture.

**Conclusions:**

Heterogeneous computing, which combines general-purpose processors with special-purpose co-processors such as FPGAs and GPUs, is a promising approach for high-performance phylogeny inference as shown by the growing body of literature in this field. FPGAs in particular are well-suited for this task because of their low power consumption as compared to many-core processors and Graphics Processor Units (GPUs) [1].

## Background

The problem of phylogenetic inference is to construct a phylogeny that most closely resembles the actual relative evolutionary history of a set of species. The species, which consist of a set of nucleotide sequences, amino acid sequences, or gene orderings, are referred to as taxa.

One of the challenges in phylogenetic inference is the size of the tree space. The number of possible unrooted phylogenetic trees for *n *taxa is:

[2]

In many cases, performing an exhaustive search to find the optimal tree is computationally intractible so heuristics are often used.

Another challenge in phylogenetic inference is determining the accuracy of a given tree. Maximum likelihood (ML) and Bayesian inference methods typically employ Felsenstein's pruning algorithm to compute the Phylogenetic Likelihood Function (PLF) in order to determine the statistical likelihood score for a tree [3,4].

This paper describes a reconfigurable hardware implementation of the Phylogenetic Likelihood Function (PLF), as well as the normalization and log-likelihood steps used in MrBayes [5]. Our design includes enhancements designed to leverage the high-bandwidth local memory on our co-processor card to store the likelihood vectors for each of the tree nodes.

MrBayes uses the PLF to evaluate the likelihood of trees [21] (which consumes nearly all of the execution time), and uses the Metropolis-coupled Markov chain Monte Carlo (MCMC) search to move through the tree space.

### Related Work

ML and Bayesian phylogeny inference tools include RAxML [6], GARLI [7], MrBayes [8], and PAML [9]. In many cases parallelized versions of these tools have been developed for cluster and shared-memory systems [10-16]. This paper instead focuses specifically on heterogeneous computing methods for likelihood-based phylogenetic inference, which requires finer-grain parallelization of the kernel computations using special-purpose co-processors.

Mak and Lam are perhaps the first team to implement likelihood-based phylogeny inference on an FPGA, but they took an embedded computing approach as opposed to a high-performance computing approach [17]. Specifically, they used the FPGA's integrated embedded processor to perform a genetic algorithm tree search method called GAML (Genetic Algorithm for Maximum Likelihood) and used special-purpose logic in the FPGA fabric to perform the PLF using fixed-point arithmetic on behalf of the software. They do not report speedups over software running on a state-of-the-art CPU, as the goal of this work was apparently to demonstrate phylogenetic inference using an FPGA-based embedded heterogeneous system-on-chip (called "platform FPGA") and not to accelerate a high-performance computer.

Alachiotis et al recently published a series of papers that describe their FPGA-based accelerator for ML-based methods [18,19]. Similar to the work by Mak and Lam, they implemented the PLF in special-purpose hardware, but their co-processor was hosted by a server running optimized C code and their PLF was double precision floating-point. In their experiments, they reconstructed trees with up to 512 taxa and achieved an average speedup of 8 relative to software on a single processor core and an average speedup of 4 relative to software on a sixteen core processor. They store the likelihood vectors, which serve as both the input and output of the PLF, in the FPGA card's local memory for high-bandwidth low-latency access. Their accelerator design also includes control logic for traversing the entire tree, reporting only the likelihood score back to the host. However, their architecture does not compute the more expensive log likelihood score, nor does it perform scaling or normalization (performed in MrBayes to prevent numerical underflow of the conditional probability vectors).

There has also been recent work in using Graphics Processor Units (GPUs) as co-processors for likelihood-based phylogenetic inference. In recent work, Suchard et al used the NVIDIA CUDA GTX280 many-core architecture to implement single and double precision versions of the PLF under a Bayesian framework using both the codon and nucleotide models [20]. Similar to Alachiotis et al, they do not compute the log-likelihood or perform scaling and normalization. Using a single computer with three GPUs, their maximum achieved speedups over single-threaded software on an Intel Core 2 Extreme where 144 for the single-precision codon model and 20 for the single precision nucleotide model (dropping to 51 and 12 for a single GPU, and 52 and 15 for three GPUs but with double precision).

### Top-Level Search

We designed our FPGA-based accelerator within the framework of the Bayesian Metropolis-Coupled Markov Chain Monte Carlo (MC^3^) method. Specifically, we used the MrBayes 3 codebase as a guide for selecting precision, identifying the computational kernels, performing the search, and to measure the baseline performance for the software case as a control for our tests.

### Single Chain Algorithm

Listed below are the main steps involved in the MCMC analysis.

(1) Given input data ***D***, randomly initialize the tree state, *s*,

(2) Propose a random move to state *s'*,

(3) Calculate the acceptance probability *P *for *s'*, according to Equation 1 below,

(4) Choose a random number between 0 and 1. If the number is less than *P*, accept the proposed state, *s *= *s'*. Otherwise maintain the old state.

(5) At user-specified intervals, "sample" the tree by recording all relevant information about the current state *s*.

(6) If the iteration count is less than the target number, go to step 2.

(7) The tree that has been accepted the greatest number of times is considered to have the greatest posterior probability (i.e. the consensus tree(1)

### Likelihood Calculation

The most expensive component of the search involves computing the acceptance probability *P*. While *P *depends on the three different ratios, computing the new likelihood ratio is significantly more expensive than computing the prior and proposal ratios. To calculate the likelihood ratio, only the numerator, *P*(*D *| *s'*), need be computed since the denominator is the saved likelihood value from the previous iteration in the loop.

Likelihood is the probability of observing the data given a particular tree. In order to make the likelihood calculation practical, MrBayes utilizes conditional probability. If we place a virtual root somewhere inside the tree and consider leaf nodes to be at the "bottom" of a tree, the conditional probability describes that the probability of everything at or below a particular node is the product of the events taking place on both descendant lineages [21]. Therefore, by starting at the bottom of the tree and working upwards, the conditional probability of each node is found by looking only at its left and right descendant nodes. Once the root, or topmost node, is reached, the overall tree likelihood is the product of the conditional probabilities for each site--or character--in the sequence data.

## Methods

### Application Partitioning

When adapting an application to any heterogeneous computing model, the target application must be partitioned into a performance-critical portion that is executed on the FPGA and a non-performance critical portion that is executed on a general-purpose CPU. In this case, the initialization, the proposing of moves, chain swapping, sampling, and the summary of the results are performed in software by the general-purpose CPU. When computing the likelihood of a proposed tree, each internal node of the tree is processed via a post-order traversal. This computation is performed on the FPGA with minimal intervention by the host. Once the likelihood is computed, the software accepts or rejects the move, and performs chain swapping and sampling as needed.

### Kernel Design

The log likelihood function contains a loop that visits each internal node in the tree, beginning from the "bottom-most" internal nodes (that are parent to two leaves) and systematically moves toward the root. The first step is to compute the conditional probability vector (actually an *n *× 4 table, where *n *is the number of characters in the aligned input data), which is performed according to Felsenstein's pruning algorithm. Given parent node *k *and children *i *and *j*, their likelihood vectors  and , and the 4 × 4 transition probability matrices *P(i) *and *P(j)*, the likelihood of base N at position c of the parent vector  is shown by Equation 2.(2)

In our target application, the conditional probability vectors are single precision floating-point values.

After this, MrBayes normalizes the conditional probability vector, generates a new *scaled likelihood vector *called *scP*, and adds this vector to a *log scaled *vector called *lnScaler *as shown in Equations 3-5. In our target application, the *scP *and *lnScaler *vectors are both single precision.(3)(4)(5)

If the current node is the virtual root, the third step is to compute the tree likelihood. This is performed by scaling each conditional probability value by the corresponding prior probabilities for each nucleotide, *π*_A _through *π*_T _drawn from the input data. These priors are sometimes called "based frequencies".(6)

The *numSites *vector is used for compression by eliminating repeated characters. In our target application, the likelihood computation is performed in double precision.

### Log(x) Considerations

As shown in the previous section, the kernel must calculate the natural logarithm of two different variables. The first calculation is single-precision floating-point and occurs in the normalization step in Equation 4. The second is double-precision floating-point and occurs in the likelihood calculation in Equation 6. The results of our timing analysis show that, combined, these two log(x) calculations consume nearly *half *of the total execution time, making the log calculation a critical component in our design. Here we describe our use of the Chebyshev approximation to implement the log function in hardware.

### Natural Logarithm Implementation

MrBayes computes the natural log of normalized values in the interval (0,1]. Since the natural log asymptotically approaches negative infinity as *x *approaches zero, the slope also approaches infinity as x -> 0, and thus any approximation method for computing the natural log requires exponentially smaller divisions of *x *values as *x *approaches 0. Execution profiling using the input sets from our experimental results section showed no values of *x *less than 10^-32^. We therefore consider the range 10^-32 ^≤ *x *≤ 1.0].

A popular approach for implementing the natural log in special-purpose hardware is to approximate the log function using Chebyshev polynomials [22-25]. Lookup tables can also be used to approximate the log function [26], but this approach requires a substantial amount of on-chip memory. In this case, we needed this memory instead for caching the output vectors.

Chebyshev polynomials of the first kind, denoted T_n_, are important in approximation theory because their roots are used as nodes in polynomial interpolation. The polynomials satisfy the following recurrence relation:

Chebyshev polynomials are a piecewise polynomial approximation method that solves a problem by dividing the input value range into segments. Each segment is approximated with a different polynomial. These polynomials are partial sums of the Chebyshev expansion for a function *f(x)*:

In our design we use 5^th ^degree Chebyshev polynomials. We are able to compute the powers of x up to x^4 ^in two stages of multiplication, and we can add the terms together in three stages. The five coefficients for each segment are stored in BRAM on the FPGA.

**Figure 1:**
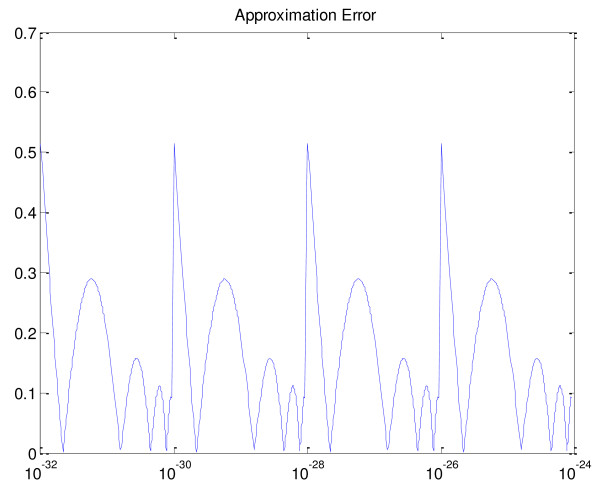
**Error Function 10^-32 ^≤ x < 10^-24^plotted over a logarithmic x-axis**. This pattern repeats over the range [10^-32 ^to 0].

As shown in Table 1, we implemented 16 segments. Figure 1 shows the approximation error over the range [10^-32^,10^-24^). While trying various log approximations, we observed that, in general, if the tree search requires the log of any *x *value less than the lower limit of the approximation, the resultant error causes the search to diverge.

**Table 1:** Segmentation for Chebyshev Approximation.

Segment	Range of *x*	Segment	Range of x
1	10^-32 ^≤ *x *< 10^-30^	9	10^-16 ^≤ *x *< 10^-14^

2	10^-30 ^≤ *x *< 10^-28^	10	10^-14 ^≤ *x *< 10^-12^

3	10^-28 ^≤ *x *< 10^-26^	11	10^-12 ^≤ *x *< 10^-10^

4	10^-26 ^≤ *x *< 10^-24^	12	10^-10 ^≤ *x *< 10^-8^

5	10^-24 ^≤ *x *< 10^-22^	13	10^-8 ^≤ *x *< 10^-6^

6	10^-22 ^≤ *x *< 10^-20^	14	10^-6 ^≤ *x *< 10^-4^

7	10^-20 ^≤ *x *< 10^-18^	15	10^-4 ^≤ *x *< 10^-2^

8	10^-18 ^≤ *x *< 10^-16^	16	10^-2 ^≤ *x *< 1.0

**Figure 2:**
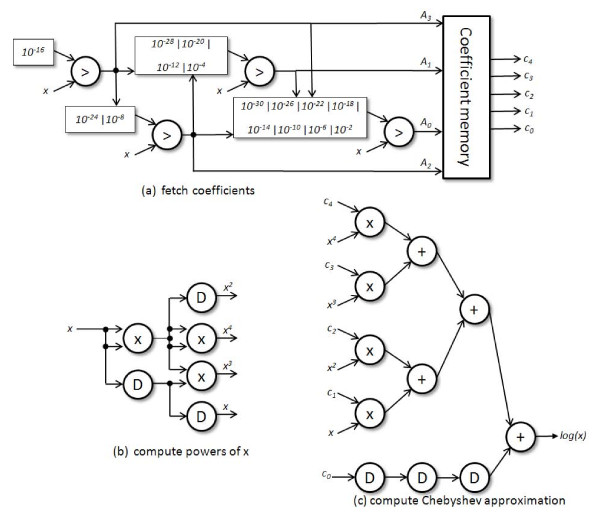
**Hardware design for Chebyshev log(x) approximation**. Shown in the figure: (a) the input value *x *is resolved into one of the sixteen sets of coefficients using a comparison network (synchronization delays not shown), (b) powers of x are computed (D blocks represent delays), and (c) the Chebyshev polynomial is computed. The total latency of this circuit is 45 cycles and 50 cycles for single- and double-precision on the Virtex-2 Pro FPGA, and 39 and 48 cycles on the Virtex-6 FPGA.

Our top-level design is shown in Figure 2. The design is composed of three components. For each incoming value of *x*, we use a radix-2 comparison network to determine the segment in which *x *falls and generate a corresponding address for the coefficient memory. In parallel to this, multipliers are used to compute powers of *x *up to *x*^4^. These values, along with the coefficient values, are eventually used to compute the Chebyshev polynomial.

We used Maplesoft Maple [27] to generate the Chebyshev coefficients and to expand the resultant polynomials. Our implementation requires four adders, seven multipliers, 20 BRAMs, and four comparators to determine which coefficients should be used for a given value of *x*. Because the Chebyshev approximation required coefficients with magnitudes greater than the upper limit for the single precision representation, we only implemented a double precision version and performed conversion in the case where the log is performed for single precision values.

### Accelerator Design

**Figure 3:**
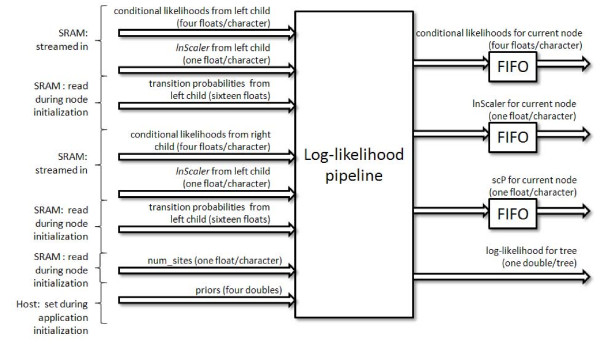
**Top-Level Design for Log Likelihood Accelerator**.

Figure 3 summaries the inputs and outputs and shows the top level design. In our target application there are three components involved in computing the log likelihood of a tree. Each step depends on the previous so they must be performed sequentially but can be parallelized using a single deep pipeline. The likelihood evaluation is only performed for the virtual root node, but in our design we combine the likelihood logic and the scaling logic, discarding the likelihood result when it is not needed by the host.

The first step in the algorithm requires two transition probability tables (two 4 × 4 nucleotide tables requiring 32 single precision floating point values), as well as conditional probability vectors for the left and right descendant nodes (eight single precision values per character). The transition probability tables can be loaded into the FPGA and maintained for each tree node, while the conditional probability vectors can be "streamed" through the pipeline on the FPGA. This component outputs a conditional probability vector for the current node (four single precision floating point values per character).

The second step requires the conditional probabilities computed in the previous step (four single precision values per character) as well as scaler values for each character (one single precision value per character) and outputs a scaled conditional probability vector (four single precision values per character), and two updated scaler values for each character ("*lnScaler*" and "*scPNew*" values--two single precision values per character).

The third step takes, as input, the conditional probability vector and scaler vector from the second step (five single precision values per character) as well as the base frequencies (four double precision values), and site occurrences (one single precision value per character), and outputs the total log likelihood (one double-precision floating point value). This design includes the logic necessary to complete the likelihood evaluation of an internal or root node, one character at a time.

### Reducing I/O

In order to make our co-processor design amenable to any phylogeny tool that requires the same log likelihood computations, we deliberately leave as much of the top-level control (i.e. the search algorithm) to the host as possible. In other words, the co-processor performs *only *the likelihood, scaling, normalization, and log likelihood computations and is not coupled to the tree search algorithm or any particular tree representation.

However, in order to minimize I/O traffic between the host and co-processor, the vectors associated with each tree node must be stored in the FPGA card's on-board (and off-chip) memory. Our FPGA card has six banks of DDR2 SRAM, giving the accelerator access to six independently addressable 72-bit memory ports per cycle (totalling 432 bits per cycle).

During initialization, the host sends the four priors (base frequencies) to the accelerator. This only occurs once per search and doesn't add any per node overhead.

Prior to processing each tree node, the host issues a programmed I/O call to the accelerator controller that indicates the unique chain/node base address for the left child, right child, and current node. These addresses correspond to memory addresses on the co-processor card's local memory and are maintained by the host. This allows the host to manage the tree topology.

After receiving this instruction, the accelerator controller loads the 4 × 4 transition probability tables for the left and right children into on-chip memory (32 floats). It also loads the current node's *numSites *array into on-chip memory. These values are loaded from two different memory ports, so the time required for this transaction is set by size of the *numSites *array.

After this, the pipeline begins processing the current node. For each sequence character, the pipeline reads four 32-bit conditional probability values and 32-bit *lnScaler *value for both child nodes per cycle. All values are read in parallel because they are distributed across three memory ports each.

During this operation, the pipeline outputs, for each sequence character, four 32-bit conditional probability, a single 32-bit *lnScaler *value, and a single 32-bit *scP *value for the current node. Since all memory ports are in use for reading, the output data must be buffered in on-chip memory until all the input data enters the pipeline. After this, the output data is written to the on board memory.

Our current design limits the computed conditional probability vectors to a size of 8192 × 4 (for each nucleotide), requiring 128 K of on-chip memory. This gives a maximum sequence length of 8192 for each of the input taxa. This limitation is imposed by difficulties in meeting timing closure for place-and-route rather than the on-chip memory capacity.

### Conditional Probability Computation

**Figure 4:**
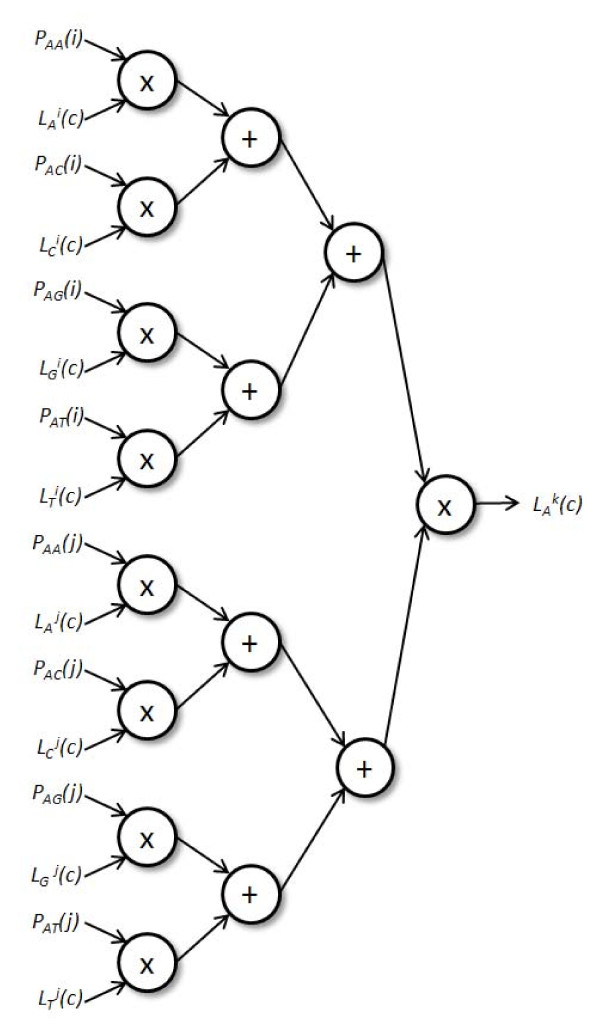
**Design for conditional probability computation**. In the accelerator design, this design is replicated four times (for each nucleotide) to implement Equation 1. The latency of this pipeline is 38 cycles on the Virtex-2 Pro FPGA and 34 cycles on the Virtex-6 FPGA, based on floating-point cores from Xilinx Core Generator.

Figure 4 shows the logic needed to update one row in the conditional probability table for the current node. This logic is replicated four times to complete the conditional probability update for one character. The complete conditional probability computation has a pipeline latency of 38 cycles on the Virtex-2 Pro FPGA and 34 cycles on the Virtex-6 FPGA, due to differences in the latencies of the floating-point units for each FPGA (the Virtex-6 has hard-IP adder components while the Virtex-2 Pro does not).

**Figure 5:**
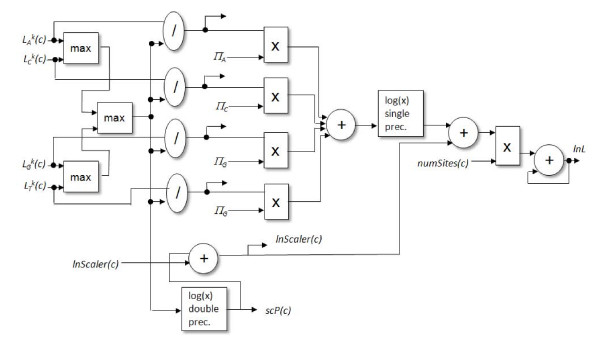
**Design for scaling and likelihood evaluation computation**. The four-input adder is implemented using a 2-stage binary adder tree. This pipeline has a total latency of 213 cycles on a Virtex-2 Pro FPGA and 227 cycles on a Virtex-6 FPGA (251 and 261 when including the conditional probability pipeline that feeds this pipeline), including single-to-double precision conversion between the normalization and likelihood pipelines (not shown).

Figure 5 shows the logic necessary to perform the scaling for the conditional probability table of a node one character at a time. The conditional probability values are provided by the conditional probability logic shown in Figure 4. The scaling step involves comparisons and divisions. The pipeline that produces the normalized conditional probabilities has a latency of 32 cycles on the Virtex-2 Pro and Virtex-6 FPGAs (not including the first stage pipeline that feeds it the un-normalized conditional probability values). The pipeline that produces the log scaler has a latency of 49 cycles on the Virtex-2 Pro and 43 cycles on the Virtex-6, or 81 cycles and 75 cycles when including the latency of the conditional probability pipeline, from which it receives its inputs.

We combine the likelihood evaluation in this block as well, although we only need to save the final value for the topmost node. The pipeline that produces the likelihood values is 125 stages deep (again not including the pipelines that provide its inputs).

### Likelihood Accumulator

As shown in Figure 5, the likelihood value must be accumulated for the root node of a tree. The figure shows a simple feedback-based accumulation circuit but this is symbolic only--double-precision addition is normally a deeply pipelined operation (having a 14 cycle latency in our case), and since new inputs arrive to the accumulator every cycle, a data hazard exists between the output of the accumulator and the next input to be accumulator. In other words, when a deeply-pipelined adder is converted into an accumulator using a feedback, the adder will accumulate α partial sums for each stage of the pipeline, where α is the pipeline depth. In this case, special logic must be used to reduce these partial sums to a final sum after all the input values have arrived.

To reconcile this problem, we have implemented a simplified version of the DSA reduction circuit developed by Prasanna [28]. Our double precision accumulator is composed of a single 14-stage double precision adder, an output buffer, and a set of multiplexers that allow the accumulator to be placed in various configurations depending on the input and output state of the adder.

Whenever the accumulator's input enable is asserted, the current accumulator input and the output of the adder are routed into the adder inputs. This means that while the accumulator is receiving a continuous stream of input values, the adder contains 14 partial sums within its pipeline.

When the accumulator's input enable is not asserted (i.e. in between likelihood evaluations), the accumulator is in a state where it coalesces the 14 partial sums. In this mode, a buffer attached to the adder output is used to capture any non-zero value that is produced by the adder. Each clock cycle where this output buffer and the adder's current output both contain non-zero values, both values are routed back into the adder and the output buffer is cleared. This process continues until all the partial sums have been reduced into a single sum, requiring five passes through the adder equalling 70 total cycles.

### Input/Output

The first time the host visits each node, data associated with the node (consisting of four conditional probability vectors, one *lnScaler *vector, one *numSites *vector, and a 4 × 4 transition probability table) are transferred to the FPGA card using direct memory access (DMA) and are stored in its local memory. Before transferring the data, the host specifies the base address that the FPGA uses for each chain and node, which are maintained in a table on the host. In other words, the host is responsible for memory allocation and management of the FPGA card's local memory, and the accelerator reads input from and writes results to addresses specified by the host. Note that while the node states (vectors) are stored in the FPGA card's local memory, the host maintains all other data associated with each tree such as the topology.

The host allocates enough space for two copies of the state information associated with each node in order to allow for a "pending" and "committed" state for each vector. After processing each node, the accelerator stores the results starting at the "pending" address. If the tree is state is committed, the host swaps the "pending" address with the "current" address for each node in the tree. If the tree is state is rejected, the host doesn't perform this swap and vectors associated with each tree node remain unchanged.

## Results

### Hardware Implementation

We designed our accelerator architecture using the Mentor Graphics FPGA Advantage CAD/EDA tools using VHDL, synthesized using Synopsys Synplify Pro 8.8.04, and placed-and-routed using Xilinx ISE 11.4.

We synthesized and place-and-routed our accelerator onto an Annapolis Micro Systems WILD-STAR II Pro computing card containing a Xirtex-2 Pro 100 FPGA and six 36-bit wide banks of DDR2 SRAM modules used for local memory. Our design operates at 165 MHz and consumes nearly all of the logic slices and hardware multipliers on the FPGA.

### Software Configuration

We used an Intel Xeon 5500-series processor to measure the software performance and act as the host for the co-processor. At the time of this writing in early 2010, the Xeon 5500-series is the most recently released and highest-performance Intel server processor available.

We compiled the MrBayes code using the gcc version 4.1.2 compiler and with the "O3" compiler optimization and with the SIMD SSE3 extensions enabled. Note that in the MrBayes code, SSE3 instructions are explicitly used for computing the conditional probability values but are not used for computing the root node's log likelihood (it is not clear why this is the case).

The default compile configuration of MrBayes uses the standard UNIX log function defined in version 2.5 of the math library shipped with Red Hat Enterprise Linux 5.4. However, MrBayes can also be compiled to use the de Soras log approximation that is included in MrBayes 3.1.2. The approximation uses the following algorithm to approximate log *x*:

1. extract the exponent from the IEEE 754 representation to obtain: ,

2. extract the mantissa *m *(where 1 ≤ *m *< 2) from the IEEE 754 representation and compute: ,

3. set 

In the MrBayes code, log 2 is approximated as the base-10 constant 0.69314718. This approximation is only used for the scaling operation and is not used for computing the log likelihood for the root node of the tree (the UNIX log function is still used for this). In the implementation of this approximation, a 0 is returned for any input values that are < 10^-10. ^Comments in the MrBayes source code states that this approximation yields errors less than 7 × 10^-3^. However, because of the hard-coded lower limit placed on *x*, the effective error of the approximation is substantially higher when it evaluates a log of a value < 10^-10 ^(as it returns 0), which actually causes the search to diverge for many datasets.

As a result of the error introduced by calls to this approximation with ×< 10^-10^, MrBayes failed to converge for all of the datasets in our experiments (i.e. the average standard deviation of split frequencies increased to its maximum value during the search). As a result, we do not include performance results for this log approximation.

### Test Data

Table 2 describes each of the test datasets, each containing DNA sequence data and downloaded from TreeBase [29]. The table is sorted by the sequence length. We ran each of these using the base software version of MrBayes 3, using the GTR substitution model and assuming clock-constrained (rooted) trees with uniform probability density on the branch lengths. We left all other options as default.

**Table 2:** Input datasets and effects of log approximation on consensus tree.

Dataset	Taxa	Chars	Gen. for SW conv	% root nodes eval	RF distance between consensus tree from the SW (base) and consensus tree from HW
m993 [33]	63	963	50 K	3.8%	9

m1319 [34]	37	1366	100 K	6.2%	3

m346 [35]	64	1620	100 K	3.8%	7

m1038 [36]	297	2021	>500 K	1.0%	21

m1485 [37]	63	3009	100 K	3.8%	13

m4056 [38]	434	9563	>500 K	1.2%	56

m3631 [39]	191	13568	>500 K	1.7%	23

Column 4 of the table shows the number of generations required by MrBayes to converge rounded up to the nearest increment of 50,000, where we (and the MrBayes output) determine that convergence has occurred when the average standard deviation of split frequencies is < 0.1.

### Effect of Log Approximation

Table 2 also lists the number of generations required for the MrBayes search to converge for each of our sample datasets. To estimate the effect of the log approximation on the quality of search results, Table 2 also lists the Robinson-Foulds distance [30], as computed by PhyloNet [31], between the consensus tree given by the base software method (used as the model tree) against the consensus tree given by the FPGA-accelerated method. In general, these distances are equal to the typical distances between multiple runs of the same dataset in the software-only control case.

## Discussion

### Accelerator Performance

Since the hardware portion of the accelerated MrBayes is a fixed latency pipeline, the performance of our design can be derived as a function of the clock speed, sequence length, and pipeline latencies.

Our Virtex-2 Pro-based accelerator produces an output every cycle after the pipeline latency of 119 cycles for non-root nodes, which require only the outputs of the conditional probability pipeline, and 251 for a root node that requires the output from the log likelihood pipeline. These latency values change to 109 and 261 for a Virtex-6 FPGA. The average time to process a node is therefore:

(119*λ *+ *λc*)·(1 - *r*) + (251*λ *+ *λc*)·*r*, for a Virtex-2 Pro 100 and (109*λ *+ *λc*) + (261*λ *+ *λc*)·*r*, for a Virtex-6 SX 475

where *λ *is the clock period, c is the sequence length, and *r *is the ratio of root node evaluations to internal node evaluations. This ratio is nominally equal to , where *n *is the number of taxa. However, in some cases the conditional probabilities for a particular node does not need to be updated, and in these cases they are not performed. In Table 2 we report this ratio for each dataset as reported at runtime.

On our Virtex-2 Pro 100 FPGA, an FPGA which is several generations old, the pipelines and memory interface operate at 165 MHz (cycle time of approximately 6 *ns*). We have also synthesized, placed, and routed our accelerator design targeting more recent FPGA technology, a Virtex-6 SX 475 FPGA, and achieved timing closure at 310 MHz (its DSP48E blocks are designed to operate at 350 MHz and these blocks generally dictate the throughput of floating-point units for which they are used [32]). We report our results based for both clock speeds.

### Performance Results

We performed all software experiments on an unloaded machine (i.e. no other processes were running to guarantee exclusive, unshared access to the processors and cache).

Table 3 lists our performance results. For each dataset we report the average CPU time required to compute a single non-root node and root node, as well as the average over all nodes. We also report the average pipeline times for each node for the FPGA implementations and corresponding speedups. As shown, we achieve a near 10× improvement over software for the 310 MHz version of our design without sacrificing the quality of the consensus trees from the search.

**Table 3:** Performance Results for SSE3 and fast log approximation.

	Average SW node processing time (μs)			Average HW node processing time (μs) and speedup relative to SW			
**Dataset**	**Non-root**	**Root node**	**Average**	**Ave. HW time/node @ 165 MHz (μs)**	**Speedup vs. SW**	**Ave. HW time/node @ 310 MHz (μs)**	**Speedup vs. SW**

m993	16.1	43.1	**17.1**	6.6	2.6	**3.5**	**4.9**

m1319	20.7	54.2	**22.7**	9.0	2.5	**4.8**	**4.7**

m346	41.1	118.0	**44.0**	9.6	4.6	**5.1**	**8.7**

m1038	46.3	119.4	**47.0**	13.0	3.6	**6.9**	**6.8**

m1485	61.5	166.2	**65.5**	19.0	3.4	**10.1**	**6.5**

m4056	193.6	560.3	**198.2**	58.7	3.4	**31.2**	**6.4**

m3631	199.6	563.9	**205.9**	83.0	2.5	**44.1**	**4.7**

## Conclusions

We successfully implemented an accelerator to MrBayes and characterized its performance. Our accelerator design exploited fine-grain parallelism using a custom, deep pipeline for computing the likelihood of a tree node. This technique can be trivially scaled up by assigning a separate FPGA in a multiple-FPGA system to each chain. This work demonstrates both the potential for accelerating Bayesian inference.

## Competing interests

The authors declare that they have no competing interests.

## Authors' contributions

SZ performed the run-time analysis of MrBayes to determine which components of the application to perform on the co-processor, designed the accelerator architecture, and wrote the bulk of the manuscript text. JDB assisted SZ in choosing the application, analyzing its runtime behavior, and designing the accelerator architectures. JDB modified the accelerator architecture so it would fit on the FPGA, added control logic to reduce the design's I/O bottleneck by caching node data in on-board memory, designed the accelerator architecture's host interface, modified the MrBayes source code to interface it to the accelerator, performed the tests to characterize performance and accuracy, edited and revised the manuscript, and performed additional synthesis runs for a more recent FPGA device. Both authors read and approved the final manuscript.
